# Involuntary Celibacy: A Review of Incel Ideology and Experiences with Dating, Rejection, and Associated Mental Health and Emotional Sequelae

**DOI:** 10.1007/s11920-022-01382-9

**Published:** 2022-11-17

**Authors:** Brandon Sparks, Alexandra M. Zidenberg, Mark E. Olver

**Affiliations:** 1 Department of Psychology, Kingston University London, 55-59 Penrhyn Rd., KT1 2EE Kingston Upon Thames, UK; 2 King’s University College, London, Canada; 3 University of Saskatchewan, Saskatoon, Canada

**Keywords:** Incel, Involuntary celibacy, Dating, Loneliness, Misogyny, Violence against women

## Abstract

**Purpose of Review:**

Incels (involuntary celibates) have recently garnered media attention for seemingly random attacks of violence. Much attention has centered around the misogynistic and violent discourse that has taken place in online incel forums as well as manifestos written by incels who have perpetrated deadly attacks. Such work overlooks the experiences and issues faced by incels themselves, the majority of which have not engaged in any violent behavior.

**Recent Findings:**

A small number of studies have recruited incels. Results from these studies highlight the nuanced nature of the incel identity. It is also apparent that incels suffer from high levels of romantic rejection and a greater degree of depressive and anxious symptoms, insecure attachment, fear of being single, and loneliness.

**Summary:**

Incels report significant issues pertaining to their mental, social, and relational well-being and may seek support from forums that often feature misogynistic and violent content.

## Introduction

### Incels, Violence, and the Media

In the evening hours of August 12, 2021, a media firestorm took place in Plymouth, England, when news emerged that a young man had shot and killed six individuals, including himself [1]. The attack was newsworthy for multiple reasons: it was the first deadly mass shooting in the UK in over a decade [2]; the act was carried out by the owner of a legally owned firearm in a country with strict gun control regulations; and the killer, Jake Davison, first shot his mother before shooting people at random in the street—including a 6-year-old girl. Left without a clear motive, much attention circulated around the fact that Davison was a self-proclaimed incel, a portmanteau of involuntary celibate [3, 4]. Incels have been responsible for a number of high-profile attacks that have resulted in the deaths of over fifty individuals [5]. This includes what has been dubbed the Toronto Van Attack where 25-year-old Alek Minassian mowed down pedestrians on a busy Toronto street with his rental vehicle, becoming one of Canada’s most deadly attacks [6]. In the leadup to the attack, Minassian publicly praised “supreme gentleman” Elliot Rodger on his social media accounts, referring to an individual who went on a shooting rampage in Isla Vista, California, killing six before committing suicide. Rodger has become a celebrated figure and martyr among the incel community for committing the first known incel-based attack and is frequently cited by other incels as serving as an inspiration for their own acts of violence [7–10].

Due to the nature of the attacks, there has been debate over whether incels should be considered a terrorist group [10, 11]. In 2020, Canada became the first country to lay terrorism charges against an incel when authorities discovered that the 17-year-old who stabbed two women (one fatally) subscribed to incel ideologies (although they did not do so with Minassian) [12]. Following the charges, the Canadian Security Intelligence Service amended its terrorism definition to incorporate a wider range of extremist political, religious, and ideological beliefs. While UK authorities did not deem the Plymouth shooting as a terrorist act, two other trials involving individuals affiliated with the incel movement are being investigated as such [2, 13]. That incels both lionize and chastise the perpetrators of these violent offenses in their online forums makes it difficult to attach the terrorism label as it does not align with more traditional, organized terrorist groups that collectively organize such attacks [11, 14]. Others claim that the incel ideology is simply an amplification of current societal beliefs and that use of the terrorist label is both erroneous and counterproductive [15].

This article serves as a review and synthesis of the extant literature about what is known about the psychology of incels, an understudied group in human sexuality and psychopathology. We begin with an overview of incel ideology, one that is marked by the language of violence and extremism, appearance-based hierarchies, and fueled by distorted perceptions of women and their sexuality. We proceed to discuss recent findings regarding incel experiences with dating and rejection and associated mental health and emotional sequelae followed by a review of methodological issues and challenges conducting research with the online incel community. We finish with conclusions and directions for future research.

### Incel Ideology: An Overview

As the name suggests, “involuntary celibates” refers to a loose collection of individuals who are experiencing sexlessness despite their desire to be active. The term dates back to 1997 when a sexually frustrated university student named Alana created a website to document her struggles in the dating sphere [16, 17]. Alana soon discovered that she was not alone in her frustration and *Alana’s Involuntary Celibacy Project* became a hub for individuals with similar experiences. The site became an informal support group of sorts that featured discussion forums, article sharing, and a mailing list, which served a diverse community of people across the age span, genders, and sexual orientations [18, 19]. Alana was ultimately successful in her dating (and mating) ambitions and left the website in the hands of other members; it was not until the Rodger attack that she discovered the group had splintered into male-only assemblies whose sexual frustrations were often directed at the women who have “shunned” them [16]. Of note, this rise of second wave incels has coincided with a decline in sexual activity among American youth in general [20], suggesting that modern-day youth are experiencing rates of sexlessness that is not in accordance with their predecessors or sociocultural tropes of hypersexual teens.

#### Violence and Extremism

As violence has served as many individuals’ introductions to incels, much of the exploratory research on incels has centered around violent ideologies in an attempt to draw a parallel between these and the acts committed by individuals such as Minassian and Rodger [21•, 22, 23]. Such work has largely been conducted by analyzing message threads on online incel forums, such as Incels.co and the now defunct subreddit r/Braincels, which was part of Reddit’s purge of incel forums due to the hostility of their content [24, 25]. These forums have admittedly made it easy to characterize incels as violence-endorsing, misogynistic, anti-feminist, and belonging to a greater “manosphere” movement that also includes Men’s Right Activists, antifeminists, and pickup artists [26, 27].

#### An Appearance-Based Hierarchy

One of the fundamental tenets of incel ideology is the belief that society is organized along a looks-based hierarchy that dictates much of social (and sexual) relations [21•]. At the top of the structure are the most highly attractive men and women, referred to as “Chads” and “Stacys,” respectively, in incel parlance. The “Alpha” status that is ascribed to these individuals is much sought after among individuals both for themselves and the people they date. This is particularly the case with “normies,” who occupy the middle and most populous tier in the hierarchy. The bottom rung of the social ladder is occupied solely by incels, a classification that is exclusive to males despite its gender neutral origins ([28] although some would disagree, see [29] for a discussion on femcels). Those occupying the upper echelons are the subject of both scorn and envy by incels; they lament Chads for hoarding all of the women while also wishing to attain this status [21•, 30, 31•].

Stacys, on the other hand, are the objects of incels’ sexual desires, but are repeatedly dehumanized in incel forums as being superficial, daft, selfish, and manipulative [21•, 31•]. The discourse surrounding females (and Stacys in particular) is the main driver in incels being viewed as misogynistic. This is not entirely unwarranted, as such discussions seem to be a staple of incel discourse. Incels appear to be fixated on a notion of female “hypergamy” wherein women are chastised for being sexually selective to the exclusion of incels (and most “normie” men). Inferred in this is that women are to blame for the looks-based societal hierarchy that has, in effect, created incels in the first place [21•, 30, 31•]. This is predicated on two beliefs: the first is that women are only interested in their own advancement and thus exclusively seek out men of higher social standing (i.e., Chads) to date. If such a male is not available, women will settle for a lesser-attractive person (i.e., a normie) who has material wealth that they can exploit to get ahead. Forever in search of the “holy trinity” of money, sex, and power, women are also seen as generally untrustworthy and prone to infidelity, willing to take any chance they can to ascend the social ladder and abandon normie men in pursuit of greener pastures [31•]. This view of women’s actions as being the result of a prescriptive algorithm operating on a narrow set of criteria contributes to the second belief that women are shallow and unable to appreciate some of the qualities (e.g., humor, intelligence) that may be found among the men who occupy the lower ranks of the social hierarchy [21•, 30, 31•].

#### Women and Sexuality

Incels often cite peer-reviewed research articles as lending credence to the tenets of their theories, with the Incels Wiki devoting a considerable amount of space to said research [32•, 33, 34]. For instance, incels have cited Rhodes [35] to indicate that women find facial symmetry more attractive and Dun and Hill [36] to provide evidence that wealthy men are perceived more favorably by women. What is missing in these discussions is a holistic view of such research studies; these factors may make someone a more favorable dating partner, but it is not an exhaustive list of traits and characteristics that women may seek. Interpreting these studies in such an absolute way causes a panic among the incel community, who feel that they do not possess these attributes [21•, 30, 37•]. The notion that women are supposedly dictating the course of the so-called sexual marketplace is highly problematic. As it was feminism that promoted and encouraged women to have a deserved right to sexual agency, there has been much discussion in incel forums dedicated to the reversal of gender equity [21•, 38, 39]. Many of the proposed solutions involve some form of coercion, rape, or a complete return to enforced monogamy under strict patriarchal rule [21•, 40]. Such highly concerning comments are imbued with feelings of sexual entitlement, which incels do score higher on than non-incel males [37•].

In the incel community, one’s ideology is reflected in what color pill they have supposedly taken. This is a metaphorical reference to the movie *The Matrix* in which the protagonist, Neo, is given the option between taking a blue pill or a red one [21•, 26]. If Neo takes the former, he will experience an artificial state of blissful ignorance. In taking the red pill, he would be exposed to the cruel realities that are inherent in life. Incels considered their unique insights into the dating world as a product of being “RedPilled.” Such wisdom is lost on Normies and Alphas, who are seen as subscribers to the “BluePill” ideology. In adopting a so-called RedPill ideology, incels are also put in a position to game the system to be seen as a more desirable mate, since they believe themselves to hold the (short) playbook that women abide by. This has led to self-enhancement practices referred to as looksmaxxing and gymmaxxing where individuals undergo plastic surgery or rigorous exercise routines in order to improve their appearance [30, 32•, 41]. This is viewed as the only solution (aside from a return to enforced monogamy) available to incels to achieve Alpha or Chad status.

Recently, a new ideology has taken hold that has been dubbed the BlackPill. It is similar in many ways to the tenets of the RedPill perspective with the added caveat that one cannot transcend the boundaries of the looks-based hierarchy [38, 42]. Incels will remain incels, normies will remain normies. If the RedPill ideology is seen as pessimistic, then the BlackPill is its nihilistic successor that promises no hopes of upward mobility unless the looks-based hierarchy collapses and a new (patriarchal) order is established. A recent survey indicated that roughly 95% of incels believe in the BlackPill, although less than half feel this is a requirement of being an incel [28]. In fact, aside from being male, there was no universal agreement among incels on who an incel is; incels varied as to whether being a virgin was a criteria for being an incel or whether a period of sexual inactivity was sufficient. Only two-thirds believed incels must be aged 18, while one-third did not believe social exclusion was a criteria, and one-quarter did not endorse physical unattractiveness as a mandatory requirement. Thus, aside from being sexually inactive males, the boundaries of inceldom are not clearly defined. They do, however, tend to reside in industrialized Western nations: in Speckhard et al.’s study [28], 32% of incels resided in Western Europe, 31% in North America, and 14% in Eastern Europe, with lesser representation in Asia (10%), Central or South America (8%), Africa (2%), and Oceania (2%).

### Dating and Rejection

There is emerging evidence that incel activity may be an active response to the local dating market. Brooks and colleagues [43] analyzed over 321 million tweets posted between 2012 and 2018 and found that areas with a greater male:female sex ratio (indicative of greater competition for mates) had a greater volume of incel-related tweets. Incel tweets were particularly high in areas that paired competitive sex ratios with fewer single women, high income inequality, and lower gender income gaps. A lack of opportunity has also been identified in a recent study on incels’ dating app experiences. For instance, despite being more liberal in their selection (opting for wider age ranges and geographic radius, swiping right on a larger percentage of people), incels reported matching with only 4.5% of individuals compared to non-incel men who reported matching with roughly one-third of individuals [44]. When matches do occur, allowing users to communicate with one another, incels reported not receiving a response 75% of the time, nearly twice the rate of non-incel men. This aligns with a large discrepancy in the frequency of positive dating app outcomes: prevalence rates of going on dates, being in a relationship, and having sex with someone met through a dating app were 33%, 0%, and 13% among incels compared to 62%, 29%, and 58% for non-incel males, respectively. Thus, rather than being liberating, incels’ dating app experiences have been marked by disappointment.

### Mental Health and Emotional Sequelae

What compounds this further is that incels report being more sensitive to rejection than their male counterparts, and experience a greater fear of being single, and that their self-esteem (which is also much lower) is more heavily influenced by their relationship status [44]. Their lack of popularity on dating apps has also been associated with higher levels of depression and dating anxiety and lower levels of self-esteem and secure attachment, all of which incels report higher and more problematic levels of than non-incel males. In a recent survey, the prevalence rate of depression and anxiety among incels was 95% and 93%, respectively, trumping national figures (gathered by the Center for Disease Control and Prevention) of 28% and 36%, respectively [45]. Formal diagnoses (38% for both) were also higher than the national averages. How incels respond to their celibate situation has recently emerged as an area of interest for researchers. Three rhyming domains have been identified based on incel slang: hope, cope, and rope [39, 46, 47]. In the latter, rope references suicide by hanging, but is shorthand for suicide by any means. It has become a prevalent enough discussion in incel forums that Daly and Laskovstov [48] were able to conduct an analysis of 80 incel suicide posts. In some instances, incels were encouraged by one another to follow through on their suicidal ideation. There is even a forum on the incels.is website called *Suicidefuel*, which was explored by Laplante and Boislard [47]. Their analysis found that rope posts tended to be characterized by feelings of despair and hopelessness. Incels reported feeling incompetent and failing in their endeavors to improve themselves or their situation. Several researchers have explored how incels cope with their sexless status and a pattern has emerged wherein incels are engaging in either solitary (reading, watching TV, lifting weights) or concerning (using drugs, consuming pornography) practices [30, 47, 48]. Most recently, Sparks et al. [37•] compared the coping strategies employed by incels to their male peers. A similar pattern emerged wherein healthier coping mechanisms (e.g., positive reframing, seeking emotional support) were more commonly practiced among non-incel men, while incels reported higher levels of problematic strategies such as behavioral disengagement and self-blame. With respect to romantic rejection, incels engaged in more self-critical rumination as well as externalization of blame than non-incels [37•]. With the former also being related to higher levels of depression and anxiety, it appears as though incels engage in both internalizing and externalizing behavior, although the latter had the smallest effect size compared to the internalizing behaviors when incels were compared to their non-incel counterparts.

Perhaps one of the most overlooked aspects of inceldom is the social isolation they experience. In the largest survey of incels conducted to date, the moderators of incels.co found that only one-third of the nearly 300 respondents reported that they had at least one friend [49]. In Maxwell et al.’s [31•] analysis of r/Braincels, loneliness emerged as one of the more prominent themes. One post included by the researchers noted that “incels aren’t just after sex… what they really want is affection and a genuine emotional bond… some say they would be happy if they could just have platonic love instead of romantic love” (p. 1864). Jaki et al. [50] also identified social isolation as a core characteristic of inceldom. The lack of social connections may help explain why so few incels (18%) reported having pictures with others on their dating app profiles relative to non-incel men (52%), the only picture category where such a sizeable difference emerged [44]. More importantly, loneliness has been emphasized in the manifestos of multiple incels who have perpetrated deadly attacks [51–53] and was mentioned in a video by Davison a month before his attack in Plymouth [4].

In lacking friends, incels are deprived of a natural outlet to express their frustrations and receive emotional support, which could help buffer against the pains of romantic rejection [54–56]. Indeed, a recent survey of incels found that they experienced significantly more loneliness than non-incel males as well as less social support from friends and family, which were both associated with greater symptoms of anxiety and depression as well as self-critical rumination [37•]. This aligns with Jones’ [30] analysis, wherein discussion of loneliness was often housed within larger conversations of mental health struggles. While the incel label carries with it negative connotations, making some wonder why individuals who did not fit those descriptions would seek them out in the first place, the need for social interaction and a sense of belongingness may help answer this question. In both Maxwell et al.’s [31•] and Daly and Laskovstov’s [48] thread analyses, incels expressed gratitude to one another for allowing them to share their struggles and for serving as a support group. The ability to share their experiences with an ever-present group may also explain why incels reported greater use of the venting coping mechanism compared to non-incel males in Sparks et al. [37•]. That these communities may serve positive, cathartic functions (much like those that Alana envisioned years ago) is an important consideration for researchers and journalists, who often describe them as hateful echo-chambers. Indeed, 58% of incels surveyed indicated that incel forums made them feel less lonely with even greater proportions reporting a sense of belonging (70%) and feeling understood (75%) as positive outcomes of forum use [28].

### Why Do Some Incels Commit Violence?

To date, there is no conclusive account or explanation for why select incels decide to engage in acts of violence; perhaps this is what makes the incel community so concerning to policy makers, feminist researchers, and the general public. For instance, while some incels lionize the perpetrators of violent attacks [28], low levels of endorsement for three high-profile incel attackers (Rodger, Minassian, and Chris Harper Mercer) were found in a recent survey of incels [45]. Another survey by Speckhard et al. [28] found that only roughly one in ten incels admired any of the above three incels. Incels themselves report varying levels of violent thoughts: scoring around the scale midpoint in one study [45], while one-quarter of respondents in another reporting occasionally having violent thoughts toward others [28]. Across both samples, however, incels reported themselves as relatively benign and as not posing much danger. Furthermore, incels overwhelmingly disagreed that incel groups endorse violence [28]. This may appear at odds with the volume of openly misogynistic and aggressive forum posts that have been analyzed by a number of researchers, but as Jaki et al. [50] concede, these may reflect efforts to enhance one’s own status in an echo chamber rather than a desire to promote violence. Regardless, it is of obvious importance to be able to separate the wolves from the sheep as it pertains to violent and non-violent incels.

Lankford [57] proposed a sexual frustration theory of aggression that is based on traditional frustration-aggression models. It is comprised of three forms of sexual frustration: (i) unfulfilled sexual desires, (ii) partner unavailability, and (iii) unsatisfied sexual activities. It is possible that all three may be relevant for understanding incel aggression. By name alone, it is apparent that incels experience frustration due to the disconnect between their sexual desires and their level of sexual activity; building on work that has tested general frustration-aggression theories, Lankford [57] notes that aggression may be amplified when sexual unfulfillment is viewed as unjust, damaging to one’s self-efficacy, or the fault of another person. This is a proverbial hat trick for incels, as they score more highly than non-incels on measures of sexual entitlement [37•], experience greater mental health issues as rejection increases [44], and hold hostile attitudes toward both the women for rejecting them [21•, 37•, 50]. With respect to unavailable partners, Lankford proposes that aggression may be more severe based on how much value is ascribed to having sex with a particular person, when they experience envy of another person for their sexual activities, or when they feel they cannot have sex with a desired person due to their own poor qualities. Again, there are parallels with the incel community; incels report greater fears of being single [37•, 44] and demonstrate animosity toward Chads [30, 31•]. They also believe that they are unattractive and belonging to a lower social class [21•], both of which were specifically identified by Lankford [57] as contributing to hostile behavior. Less is known about whether incels feel dissatisfied with their sexual activities; gauging incels’ use of pornography and masturbation may be beneficial to future researchers for the reasons posited in Lankford. Lankford identifies four means (relief-seeking, power-seeking, revenge-seeking, and displaced frustration) by which individuals may seek to relieve themselves of their sexual frustration. With respect to incels, the revenge-seeking may be the most relevant as it is most closely associated with holding misogynistic attitudes (which a number of researchers noted above have associated with incels) and individuals such as Rodger [53] referred to their violent acts “retribution.” Furthermore, misogynistic attitudes significantly predicted violent ideations, willingness to commit rape, and their perceived dangerousness in a sample of incels [28]. While this model is useful in understanding why incels may be more prone to aggression, it alone cannot explain why only a small number of incels have carried out violent acts. Similarly, Williams and Arntfield [23] identified sexual frustration, social isolation, family problems, and employment instability as contributors to seven incel attacks, yet these are issues that plague the incel community more generally [37•, 58].

A recent systematic review of 78 studies identified nine risk factors for lone-wolf attacks, under which they classify violent incels [59]. These nine are (i) sociodemographic characteristics, (ii) social ties, (iii) interpersonal rejection, (iv) mental illness, (v) subclinical personality traits, (vi) strain, (vii) grievances, (viii) emotional traits/states, and (ix) cognitive processes. This, too, makes it difficult to differentiate violent from non-violent incels, as incels experience a great deal of both social and romantic rejection, isolation, and high rates of mental duress [31•, 44]. An argument could also be made that their fear of being single, rejection sensitivity, and relationship-contingent self-esteem also make them prone to strain and negative emotional states, while incel forums offer many examples of grievances [30, 31•, 42]. Moskalenko et al. [45] developed an incel radicalization scale, which has demonstrated some convergent validity with other measures of radical action [60].

All of this may have implications for the propensity for problematic sexual behavior by incels, such as sexual violence and coercion. Rejection, loneliness, distorted thinking, attitudes of sexual entitlement, and hostility toward women are well established correlates of sexual aggression and recidivism [61], and by extension, risk-relevant propensities such as these could well manifest in sexual aggression. Sexual violence in this sense would represent a fusion of sex and aggression per Lankford, in which case, women are dehumanized and sexual violence both serves as a source of sexual gratification and outlet for the individual’s frustration, rage, and aggression. Research has yet to examine the sexual violence risk profiles of this population or how such dynamics may manifest in sexual violence toward women.

Although these theories are unable to discriminate between violent and non-violent incels, they do suggest that incels may be an at-risk group for harm to themselves or others. Given that Rodgers and Minassians of the incel community make up an incredibly small proportion, it is important to delineate inceldom from terrorism. While research should continue to help distinguish which incels may be at the highest risk of violence, efforts may be better spent trying to reduce some of the issues faced by incels, rather than seeking out a holy grail.

### Researching the Online Incel Community: Issues and Challenges

As noted above, much of the knowledge base that we have on incels is built upon analyses of online forum discussions. In order to make these analyses feasible, selection criteria had to be employed (for instance, at the time that Jones [30] began their analysis, Incels.co had over 100,000 threads). Some authors (e.g., [26, 31•, 38]) opted to use popularity-based metrics, choosing the threads based on the number of positive votes received or comments in response to the initial post. Such criteria are common means of analyzing online discourse; however, they run the risk of capturing the most inflammatory and controversial threads that may not be representative of more mundane incel communications [32•, 62, 63]. This is further compounded by the prevalence of “shit-posting” that takes place in incel forums, where users will create provocative posts to deliberately elicit strong reactions [32•, 64, 65]. In the only known study to interview incels, one forum moderator lamented that these inflammatory posts tend to quickly dominate discussions and become highly popular before being taken down for violating community rules (if indeed there are any) [32•].

Other researchers (e.g., [30, 42, 50]) have opted to use chronology-based inclusion criteria, capturing threads that were posted during a set time period. While advantageous, it also runs the risk of being skewed by real-world events that take place, such as the Toronto Van Attack, which resulted in a huge uptick in activity during Jaki and colleagues’ [50] timeframe. As O’Donnell and Shor [22] note, much of this dialog was in support of Minassian’s self-described “rebellion.” Another important consideration for both researcher camps is that content is disproportionately generated by a small pool of users. When Baele et al. [21•] analyzed posts on the incels.me website (the precursor to incels.il), they reported that only 46% of the 5172 members were active posters, averaging 1.6 posts per day. The top fifty posters, however, posted at a five-fold rate, with 19 users averaging just under twenty posts a day. This is an important consideration not only because it highlights that analyses may reflect the activity of a small number of users, but also because numerous studies have found that posters tend to score higher on Dark Triad personality traits than lurkers (i.e., those who do not contribute original content) [66, 67]. Thus, the above research is perhaps best described as reflecting the incel community and the discourse that takes place in it rather than the individual characteristics and experiences of incels themselves.

In the same vein, recent studies that have recruited incels do suggest that these qualitative analyses are not necessarily inaccurate. Large effects have been found in incel endorsement of Rogers et al.’s [68] Belief in Female Sexual Deceptiveness Scale vis-à-vis their non-incel counterparts [37•]. The scale, which asks questions such as “women marry wealthy husbands, but cheat with younger, better-looking men” and “women enjoy toying with men’s feelings,” aligns quite closely with the discourse of women that has been reported on in the above studies. In fact, while recruiting for this study on Reddit, one user specifically mentioned how much they enjoyed filling out this particular set of questions, describing it as “pure gold.” Across multiple studies, incels have also demonstrated a pattern of having low attachment security while scoring higher than their non-incel peers on both anxious and avoidant attachment [37•, 44]. These findings suggest that the hostility and distrust of women that have been reported in incel forums may in fact be reflective of the attitudes and beliefs held by incels themselves. Whether other findings are consistent with forum thread analyses remains to be seen; the authors caution readers not to be prescriptive when hearing the incel label. When recruiting for one of our studies, one incel commented that we may be “surprised by the lack of propensity for violence” (although this was not being gauged in said study), suggesting that some stereotypes may not reflect the entire incel cohort. Daly and Reed’s [32•] interviews further the cause to use discretion when conducting incel research or working with this population more generally. For instance, they noted that contrary to expectation, their incel participants were not preoccupied with using anti-feminist rhetoric (except when discussing the practice of shit-posting) and were more interested in sharing their lived experiences with the interviewer, who it was noted was female. While it is possible that the gender of the interviewer may have factored into incels’ decisions to participate and the content they opted to discuss, it suggests that the openly misogynistic and anti-woman dialog that takes place on incels forums may not represent all, or even a majority, of incels. Furthermore, it suggests that at least some incels do not oppose female researchers and are even willing to support their scholarly ambitions.

## Conclusions

Incels represent a high-need group with respect to clinical symptoms and overall well-being. They experience considerable duress on account of their singlehood, report that their (low) self-esteem is contingent on their relationship status, and are both sensitive to rejection and experience a large degree of it [44]. They cope with this rejection by engaging in both inward and outward practices, reporting higher levels of self-critical rumination and externalization of blame, while also engaging in fewer healthy coping mechanisms [37•]. Incels also report high rates of depressive and anxious symptoms and diagnoses as well as high rates of autism spectrum disorder [37•, 44, 45]. Furthermore, incels are plagued with low attachment security, loneliness, and a lack of social supports [31•, 37•, 44]. There is also evidence of sexist attitudes in their forums, which incels admit may be making them more misogynistic, of which there has been some quantitative evidence [28, 37•]. It is needless to say that more research on incels is needed in order to fully capture their personal experiences and relational struggles. Such efforts are obstructed by a strong distrust of academics [32•]; for instance, when recruiting for Sparks et al. [44], the first author received several threatening or concerning responses. Incels also equate universities with feminist causes and as such are wary to participate in research. During recruitment for another study, we were asked by an incel whether it would adopt a pro-feminist approach or a “sympathetic and supportive” one, suggesting that the former is seen as adversarial to incel experiences. Researchers must be cognizant of these beliefs and work to ensure that their work does not further alienate this group.

### Future Directions

A recent article by Leroux and Boislard [69] highlights the dearth of research exploring the difficulties faced by both male and female virgins during emerging adulthood. Those interviewed by the authors reported some familiar themes expressed by incels, namely feelings of loneliness, sadness, jealousy, frustration, fear, and a belief that others (including mental health professionals) may not understand their situation. Earlier work by Donnelly et al. [70] also reported some overlap between adult virgins and incels; 89% of male virgins in their study reported shyness while 41% of virgins overall reported an inability to relate to others socially. This is consistent with incels’ higher rates of dating anxiety and of ASD diagnoses, which may make social connections more difficult [44, 45]. With prolonged virginity and involuntary celibacy more broadly being underdeveloped areas of research, there is much work to be done to better understand modern-day incels who share some commonalities with their sexually inexperienced brethren, but also present unique challenges.

One of the most pressing questions that research has yet to address is how individuals gravitate toward and later identify as an incel. Those interviewed by Daly and Reed [32•] suggested that many of the adverse emotional sequelae associated with incel status (e.g., depression, loneliness) were the product of being an incel rather than contributing factors, although more work is needed here. At the other end of the spectrum, a new subreddit has emerged, r/IncelExit, which, as the name suggests, serves as a community for individuals looking to distance themselves from their former incel associations. Understanding their transition out of inceldom may offer researchers, practitioners, and even incels themselves useful insights. Given the many intra- and interpersonal struggles reported by incels, it is vital for this work to continue to develop proper clinical interventions for incels. While some incels may view therapy as a BluePill strategy, perhaps they would be more willing to participate in a treatment program tailored to their needs (e.g., enhancing their social skills) rather than one that assumes they are simply misogynistic terrorists-in-waiting. Indeed, in Moskalenko et al.’s [45] survey, roughly half had engaged in some form of psychotherapy, with twice as many (15%) reporting that it made them feel worse than better (6%). High rates of autism spectrum disorder (ASD) have also been reported in the community [45], which may shed some light on their experiences with social isolation and offer some clinically relevant suggestions for working with this population. In order to best support this population and mitigate their potential for violence (either inward or outward) related to incel identity, more high-quality research on the population and their specific needs is warranted.
